# Prognosis of surgery combined with different adjuvant therapies in esophageal cancer treatment: a network meta-analysis

**DOI:** 10.18632/oncotarget.16193

**Published:** 2017-03-14

**Authors:** Shenglei Li, Hongtao Liu, Changying Diao, Xiaohui Wang, Ming Gao, Zongming Li, Lijie Song, Xianzheng Gao, Jing Han, Feng Wang, Wencai Li, Xinwei Han

**Affiliations:** ^1^ Department of Pathology, The First Affiliated Hospital of Zhengzhou University, Zhengzhou, Henan, 50000, China; ^2^ Laboratory for Cell Biology, College of Life Sciences of Zhengzhou University, Zhengzhou, Henan, 450001, China; ^3^ Department of Oncology, The First Affiliated Hospital of Zhengzhou University, Zhengzhou, Henan, 450000, China; ^4^ Department of Interventional Therapy, The First Affiliated Hospital of Zhengzhou University, Zhengzhou, Henan, 450000, China

**Keywords:** esophageal cancer, surgery, adjuvant therapies, chemotherapy, radiotherapy

## Abstract

This network meta-analysis was conducted to assess whether the efficacy of surgery with adjuvant therapies, including radiotherapy (RT+S), chemotherapy (CT+S), and chemoradiotherapy (CRT+S) have better performance in esophageal cancer treatment and management. PubMed and EMBASE were used to search for relevant trials. Both conventional pair-wise and network meta-analyses were carried out. The surface under the cumulative ranking curve (SUCRA) was used to rank interventions based on the efficacy of the treatment method. As for 3-year overall survival (OS), CRT+S showed the highest efficacy (CRT+S vs. surgery: HR=0.81, 95% CrI =0.73-0.90; CRT+S vs. CT+S: HR=0.82, 95% CrI =0.70-0.95; CRT+S vs. RT+S: HR=0.77, 95% CrI =0.62-0.95). For disease-free survival, CRT+S showed efficacy over CT+S ((HR =0.70, 95% CrI =0. 59-0.83). In conclusion, CRT+S showed a better performance for survival outcomes and ranks best among all therapies. The results of our study can provide guidance for medical decisions and treatment options that may help clinical practitioners improve the efficacy of EC treatment.

## INTRODUCTION

Esophageal cancer (EC) is a typical malignant tumor which is often lethal for patients [1]. It is estimated that 16,910 new cases of EC would be diagnosed in 2016 in the USA alone with 15,690 EC deaths [2]. The incidence rate of EC varies from region to region, while some regions including Asia, southern and eastern Africa exhibit a higher rate [3, 4]. Researchers suggested that EC has become one of the most severe malignant tumors in western countries and more than half of new EC cases in the US were diagnosed as adenocarcinoma [5, 6]. Smoking, alcohol consumption, opium abuse and poor dietary habits etc. have been found to be the risk factors of EC [7, 8].

Surgical resection is a common choice for patients with EC [9]. However, patients underwent surgery appeared to have higher mortality rates compared with those who with alternative treatments [10]. The efficacy of surgery are not satisfactory, as studies suggested that these patients had a median survival period of only 18 months [11]. Radiotherapy (RT) is an important option which is commonly used in patients with advanced or metastasized EC [12]. The monotherapy of RT appears to have limited effectiveness and the five-year overall survival rate is approximately 10% [13]. Chemotherapy (CT) is another important therapy for cancers, and researchers have investigated the curative efficacy of CT on EC since 1990s [14]. As suggested by previous studies, combined CT appeared to have more favorable effects compared to single-agent CT [15]. Moreover, chemoradiotherapy (CRT) has been developed as a new approach for metastasis prevention and has recently become a more popular treatment option [16].

A large number of randomized clinical trials (RCTs) have been conducted to evaluate the relative usefulness of the above-mentioned approaches for controlling EC. However, there is substantial variation in the conclusions of these investigations. For example, Ando *et al*. demonstrated that preoperative CT followed by surgery can improve the survival status significantly compared to postoperative CT [17]. Nevertheless, this conclusion was controversial, and has been challenged by different researchers [18, 19]. Although several pair-wise meta-analyses based on a large number of trials have been carried out to address this inconsistency, the lack of indirect evidence prevented researchers from comparing multiple therapies simultaneously [20–22]. Therefore, we conducted this network meta-analysis to introduce indirect evidence as a potential solution to address the limitations of accurate estimates in EC treatment. In our study, we attempted to determine the relative efficacy of surgical resections and adjuvant therapies. Using a network meta-analysis approach, we compared the efficacy of surgery alone with surgery combined adjuvant therapies RT+S, CT+S and CRT+S.

## RESULTS

### Characteristics of the studies included in analysis

**Figure 1:**
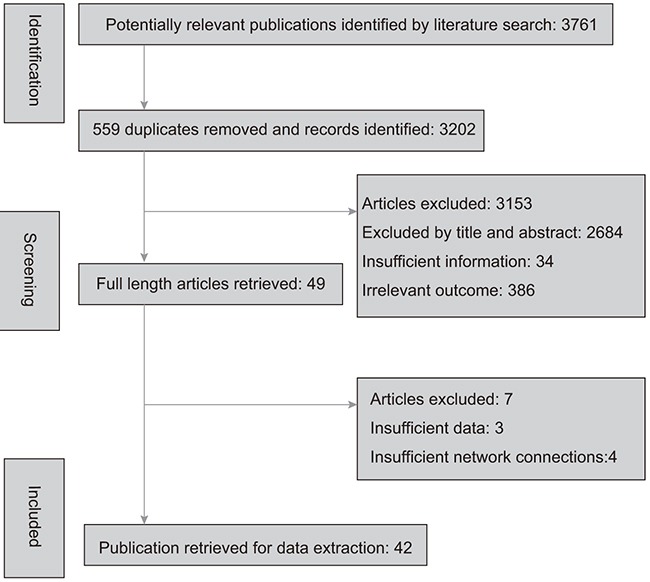
Flow chart There are 42 studies included at last.

Characteristics of all involved studies were presented in Table 1, including the original country, sample size, the intervention and control groups, histology and clinical outcomes. A detailed list of included studies, patients, and diagnostic criteria characteristics of each individual study was provided in the analyzed report. All included studies [16, 17, 23–62] were published between 1981 and 2016, and covered a broad geographic area including countries in Asia and Europe, as well as the USA and Australia, and the selection process was presented in Figure 1. The intervention group involved a total of 3,206 patients while the control group contained 3,270 patients.

**Table 1:** Main characteristics of included studies

Study or Subgroup	Country	Histology	Intervention Group			Control Group		Overall Survival		Metastasis/Recurrence	
			Size	Type	Dose (mg/m^2^)	Size	Type	Follow-up (mo)	HR and 95%Cl	Intervention	Control
Law *et al*., 1997	China	SCC	74	CT+S	C:100 d1; F:500 d1-5	73	S	17	0.73 (0.53, 1.00)	12/29	19/50
Ancona *et al*., 2001	Italy	SCC	47	CT+S	C:100 d1; F:500 d1-5	47	S	24	0.84 (0.58, 1.10)	19/28	19/29
Kelsen *et al*., 2007	USA	SCC&AC	216	CT+S	C:100 d1; F:1000 d1-5	227	S	56	1.07 (0.87, 1.32)	NR	NR
Allum *et al*., 2009	UK	SCC	400	CT+S	C:100 d1; F:1000 d1-5	402	S	37	0.84 (0.72, 0.98)	68/82	60/101
Boonstra *et al*., 2011	Netherland	SCC	85	CT+S	C:80 d1; Eto:100 d1,2	84	S	60	0.71 (0.51, 0.98)	14/25	15/31
Ando *et al*., 2012	Japan	SCC	164	CT+S	C:80 d1; F:800 d1-5	166	S	62	0.64 (0.45, 0.91)	NR/51	NR/41
Maipang *et al*., 1994	Thailand	SCC	24	CT+S	C:100 d1; Vinblastine:3 d1-4; B:10 d1-5	22	S	17	1.61 (0.79, 3.27)	NR	NR
Nygaard *et al*., 1992*	Norway	SCC	50	CT+S	C:20 d1-5; B:10mg, d1-5	41	S	18	1.10 (0.93, 1.30)	NR	NR
Nygaard *et al*., 1992*	Norway	SCC	47	CRT+S	C:20 d1-5; B:10mg, d1-5; 35GY	41	S	18	0.76 (0.45, 1.28)	NR	NR
Nygaard *et al*., 1992*	Norway	SCC	48	RT+S	35Gy	41	S	18	0.80 (0.63, 1.02)	NR	NR
Schlag *et al*., 1992	German	SCC	22	CT+S	C:20 d1-5;F:1000, d1-d5	24	S	75	0.97 (0.60, 1.57)	NR	NR
Ychou *et al*., 2011	France	AC, GEJ	113	CT+S	C:100 d1; F:800, d1-5	111	S	60	0.69 (0.50, 0.95)	49/63	62/71
Pouliquen *et al*., 1996	France	SCC	52	CT+S	C:100, d1; F:20, d1-5	68	S	NR	1.03 (0.89, 1.13)	NR	NR
Ando *et al*., 1997	Japan	SCC	105	CT+S	C:70 d1,21; V: 3 d1.21	100	S	59.2	1.08 (0.87, 1.34)	NR/57	NR/55
Ando *et al*., 2003	Japan	SCC	120	CT+S	C:80 d1; F:800, d1-5	122	S	NR	1.20 (0.96, 1.51)	NR/63	NR/45
Lee *et al*., 2005	Korea	SCC	40	CT+S	C:60 d1-4; F:1000, d1-3	52	S	25	0.60 (0.47, 0.77)	18/28	9/19
Heroor *et al*., 2003	Japan	SCC	94	CT+S	C:70 d1; F:700, d1-4, V 3, d1	117	S	80	1.46 (1.21, 1.71)	NR	NR
Shiozaki *et al*., 2004	Japan	SCC	98	CT+S	C:10, d1-5; F250-500, d1-5	52	S	NR	0.48 (0.35, 0.66)	NR	NR
Zhang *et al*., 2008	China	SCC&AC	66	CT+S	C:25, d1-3; F:375, d1-5; L:135 d1-5	160	S	NR	1.36 (0.93, 1.98)	NR	NR
Walsh *et al*., 1996	Ireland	AC	55	CRT+S	C:75; F:15mg/kg/d; 45Gy	55	S	10	0.53 (0.33, 0.84)	NR	NR
Urba *et al*., 2001	USA	SCC&AC	47	CRT+S	C:20; F:300; 35Gy	50	S	98	0.75 (0.46, 1.22)	NR	NR
Stahl *et al*., 2009	German	SCC	60	CRT+S	C:50; Eto:80; 30Gy	59	CT^+^+S	45.6	0.67 (0.41, 1.09)	NR/19	NR/27
Burmeister *et al*., 2011	Australia	AC	39	CRT+S	C:80; F:1000 /d; 35Gy	36	CT^#^+S	65	0.79 (0.41, 1.54)	NR/18	NR/21
Tepper *et al*., 2008	USA	SCC&AC	30	CRT+S	C:100; F:1000; 41.5Gy	26	S	60	0.51 (0.38, 0.68)	NR/9	NR/12
van Hagen *et al*., 2012	Netherlands	SCC&AC	178	CRT+S	Carboplatin:2mg/ ml/min; P:50 ;41.4Gy	188	S	45.4	0.73 (0.54, 1.00)	NR/62	NR/188
Burmeister *et al*., 2005	Australia	SCC&AC	128	CRT+S	C:80; F:1800; 35Gy	128	S	65	0.89 (0.67, 1.19)	48/61	54/68
Lv *et al*., 2010**	China	SCC	80	CRT+S	P:135 d1,22; C 20 d1-3 and 22-25; 40Gy	80	S	45	0.71 (0.60, 0.85)	NR	NR
Lv *et al*., 2010**	China	SCC	78	CRT+S	P:135 d1,22; C 20 d1-3 and 22-25; 40Gy	80	S	45	0.68 (0.58, 0.82)	NR	NR
Apinop *et al*., 1994	Thailand	SCC	35	CRT+S	C 100 d1,22; F: 1000 d1-4and22-25; 40Gy	34	S	NR	0.80 (0.48, 1.34)	NR	NR
Le Prise *et al*., 1994	France	SCC	41	CRT+S	C 100 d1,22; F: 600 d1-4and22-25; 20Gy	45	S	16	0.85 (0.50, 1.46)	8/17	10/17
Walsh *et al*., 1995	Ireland	AC	58	CRT+S	C:75; F:15mg/kg/d; 45Gy	55	S	10	0.58 (0.38, 0.88)	NR	NR
Mariette *et al*., 2014	France	SCC	98	CRT+S	C 75 d1; F:800 on d1-4; 45Gy	97	S	93.6	0.99 (0.69, 1.40)	22/28	28/43
Kobayashi *et al*., 2000	Japan	NR	91	CT+S	F:600	80	S	NR	1.10 (0.67, 1.81)	NR	NR
Launois *et al*., 1981	France	SCC	67	RT+S	64-90Gy preop	57	S	NR	1.17 (1.04, 1.32)	NR	NR
Gignoux *et al*., 1987	Europe	SCC	115	RT+S	33 Gy preop	114	S	43.2	1.12 (0.95, 1.32)	NR	NR
Arnott *et al*., 1992	Scotland	SCC	90	RT+S	20 Gy Preop	86	S	NR	1.02 (0.87, 1.19)	NR	NR
Lee *et al*., 2004	Korea	SCC	51	CRT+S	C:60; F:1000; 45.6Gy	50	S	25	0.88 (0.48, 1.62)	6/19	12/18

Intervention: NR-not report C - Cisplatin, F-Fluorouracil, Eto-Etoposide, B-Bleomycin, V-Vindesine, P-Paclitaxel; Treatment: CRT-chemoradiotherapy, S-surgery, CT-chemotherapy, RT-radiotherapy; Tumor: SCC-Squamous Cell Carcinoma, AC-Adenocarcinoma, GEJ-Gastroesophageal Junction; CI-Confidence Interval; HR-Hazard Ratio

Note:*: These three studies are from the same paper. **: These two studies are from the same paper. The first one is the preoperative group and the latter one is the postoperative group. +: The dose is F: 2000mg/m2, leucovorin: 500mg/m2, C: 50mg/m2; #: The dose is C:80mg/m2, F:1000mg/m2.

**Figure 2:**
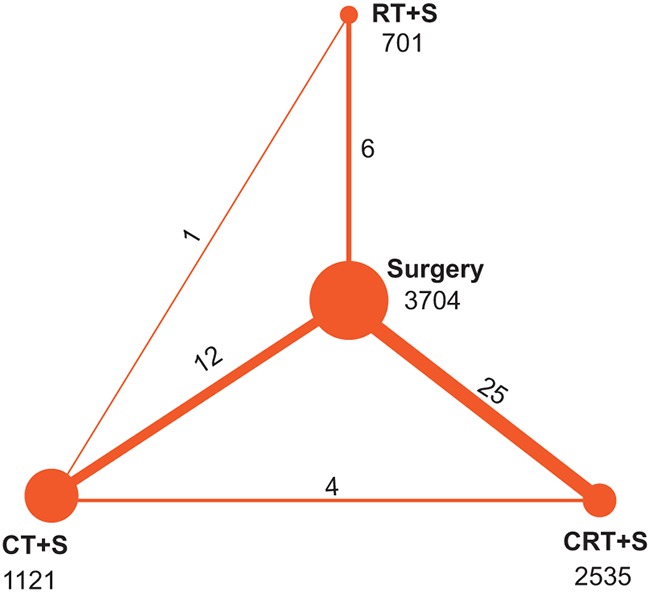
Network of randomized controlled trials comparing different treatments of EC Treatment: CRT+S-chemo-radiotherapy plus surgery, CT+S-chemotherapy plus surgery, RT+S-radiotherapy plus surgery. Numbers above lines represent direct comparisons between two treatments. Numbers above dots represent total size of the treatment.

Systematic reviews are presented in Supplementary Figure 1, indicating that no obvious publication bias was observed. A Jadad scale table was also generated and is presented in Supplementary Table 1. The width of the lines in Figure 2 represent the number of trials comparing each pair of treatments and the area of circles indicate the cumulative number of patients for each intervention.

### Pair-wise comparisons

The original data of 3-year OS, 5-year OS, and DFS are shown in Table 2. We conducted pair-wise meta-analysis and calculated the hazard ratio (HR) and 95% confidence interval (CI).

**Table 2:** Overall survival in 3 years and 5 years and disease-free survival of included studies

Study or Subgroup	Intervention			OS (HR and 95% CI)		DFS (HR and 95%CI)
	Treatment (Size)			3-year	5-year	
Law *et al*., 1997	CT+S	/S	(74/73)	-	0.73 (0.53, 1.00)	-
Ancona *et al*., 2001	CT+S	/S	(47/47)	-	0.84 (0.58, 1.10)	-
Kelsen *et al*., 2007	CT+S	/S	(216/227)	1.05 (0.88, 1.26)	1.06 (0.89, 1.25)	-
Allum *et al*., 2009	CT+S	/S	(400/402)	0.84 (0.70, 1.01)	0.84 (0.58, 1.10)	0.82 (0.71, 0.95)
Boonstra *et al*., 2011	CT+S	/S	(85/84)	0.65 (0.53, 0.80)	0.66 (0.55, 0.80)	0.72 (0.52, 1.00)
Ando *et al*., 2012	CT+S	/S	(164/166)	0.71 (0.53, 0.95)	0.67 (0.52, 0.87)	0.73 (0.54, 0.99)***
Maipang *et al*., 1994	CT+S	/S	(24/22)	1.08 (0.79, 1.48)	-	-
Nygaard *et al*., 1992 *	CT+S	/S	(50/41)	1.10 (0.93, 1.30)	-	-
Nygaard *et al*., 1992 *	CRT+S	/S	(47/41)	0.8 (0.63, 1.02)	-	-
Nygaard *et al*., 1992 *	RT+S	/S	(48/41)	0.76 (0.45, 1.28)	-	-
Schlag *et al*., 1992	CT+S	/S	(22/24)	0.97 (0.60, 1.57)	0.97 (0.60, 1.57)	-
Ychou *et al*., 2011	CT+S	/S	(113/111)	0.69 (0.50, 0.95)	-	0.69 (0.50, 0.95)
Pouliquen *et al*., 1996	CT+S	/S	(52/68)	0.99 (0.84, 1.17)	1.00 (0.86, 1.18)	-
Ando *et al*., 1997	CT+S	/S	(105/100)	0.87 (0.65, 1.18)	1.00 (0.86, 1.18)	-
Ando *et al*., 2003	CT+S	/S	(120/122)	0.77 (0.56, 1.07)	0.75 (0.56, 0.99)	0.73 (0.54, 0.99)
Lee *et al*., 2005	CT+S	/S	(40/52)	0.85 (0.66, 1.08)	0.86 (0.68, 1.09)	0.68 (0.55, 0.83)
Heroor *et al*., 2003	CT+S	/S	(94/117)	1.24 (0.94, 1.65)	1.31 (1.03, 1.67)	-
Shiozaki *et al*., 2004	CT+S	/S	(98/52)	-	0.48 (0.35, 0.66)	-
Zhang *et al*., 2008	CT+S	/S	(66/160)	1.36 (0.93, 1.98)	-	1.80 (1.26, 2.59)
Walsh *et al*., 1996	CRT+S	/S	(55/55)	-	0.53 (0.33, 0.84)	-
Urba *et al*., 2001	CRT+S	/S	(47/50)	0.75 (0.46, 1.22)	-	-
Stahl *et al*., 2009	CRT+S	/CT+S	(60/59)	-	0.67 (0.41, 1.09)	-
Burmeister *et al*., 2011	CRT+S	/CT+S	(39/36)	0.59 (0.46, 0.77)	0.63 (0.49, 0.81)	0.74 (0.53, 1.02)***
Tepper *et al*., 2008	CRT+S	/S	(30/26)	0.55 (0.40, 0.76)	0.52 (0.39, 0.70)	0.35 (0.27, 0.46)***
van Hagen *et al*., 2012	CRT+S	/S	(178/188)	-	0.73 (0.53, 1.00)	-
Burmeister *et al*., 2005	CRT+S	/S	(128/128)	0.87 (0.72, 1.05)	0.88 (0.73, 1.06)	0.82 (0.71, 0.95)***
Lv *et al*., 2010 **	CRT+S	/S	(80/80)	0.85 (0.61, 1.18)	0.83 (0.63, 1.08)	0.66 (0.55, 0.78)***
Lv *et al*., 2010 **	CRT+S	/S	(78/80)	0.73 (0.55, 0.98)	0.76 (0.60, 0.97)	0.70 (0.59, 0.83)***
Apinop *et al*., 1994	CRT+S	/S	(35/34)	0.80 (0.48, 1.34)	0.80 (0.48, 1.34)	-
Le Prise *et al*., 1994	CRT+S	/S	(41/45)	0.85 (0.50, 1.46)	0.85 (0.50, 1.46)	0.75 (0.64, 0.90)
Walsh *et al*., 1995	CRT+S	/S	(58/55)	0.58 (0.38, 0.88)	0.58 (0.38, 0.88)	-
Mariette *et al*., 2014	CRT+S	/S	(98/97)	0.99 (0.69, 1.40)	-	-
Kobayashi *et al*., 2000	CT+S	/S	(91/80)	1.10 (0.67, 1.81)	1.10 (0.67, 1.81)	-
Launois *et al*., 1981	RT+S	/S	(67/57)	1.17 (1.04, 1.32)	1.17 (1.04, 1.32)	-
Gignoux *et al*., 1987	RT+S	/S	(115/114)	1.07 (0.89, 1.29)	1.04 (0.87, 1.24)	-
Arnott *et al*., 1992	RT+S	/S	(90/86)	-	1.02 (0.87, 1.19)	-
Lee *et al*., 2004	CRT+S	/S	(51/50)	1.19 (0.92, 1.56)	-	0.98 (0.55, 1.72)

Abbreviation: OS-Overall survival, DFS-Disease-free survival, HR-Hazard ratio, CI-Confidence interval, CRT-chemoradiotherapy, S-surgery, CT-chemotherapy, RT-radiotherapy

Note: *: These three studies are from the same paper. **: These two studies are from the same paper. The first one is the preoperative group and the latter one is the postoperative group. ***: Progression-Free Survival

As shown in Table 3, the comparison between surgery alone and the combination of CT+S demonstrated that CT+S had better performance compared to surgery alone with respect to 3-year OS (HR = 0.88, 95% CI = 0.82-0.93), 5-year OS (HR = 0.81, 95% CI = 0.76-0.86) and DFS (HR = 0.76, 95% CI = 0.69-0.83). Similarly, surgery tended to present a more promising result when CRT was also involved in the treatment, with an significant promotion of 3-year OS (HR = 0.77, 95% CI = 0.69-0.85), 5-year OS (HR = 0.70, 95% CI = 0.63-0.78) and DFS (HR = 0.59, 95% CI = 0.53-0.65). On the contrary, RT+S presented a poor treatment effect compared to surgery with regards to 3-year OS (HR = 1.05, 95% CI = 0.95-1.15) and 5-year OS (HR = 1.09, 95% CI = 0.99-1.18). These statistics indicated that RT+S was unable to noticeably enhance the prognosis features of surgical treatment of EC. CT+S had a poorer performance rate than CRT+S with respect to the survival rates of 3-year OS (HR = 0.60, 95% CI = 0.44-0.75), 5-year OS (HR = 0.64, 95% CI = 0.49-0.78) and DFS (HR = 0.74, 95% CI = 0.50-0.99).

**Table 3:** Direct pairwise comparison results of esophageal cancer treatments

Comparison	3-year OS	5-year OS	DFS	Recurrence	Metastasis
CT+S vs S	**0.88 (0.82, 0.93)**	**0.81 (0.76, 0.86)**	**0.76 (0.69,0.83)**	0.85 (0.73, 1.00)	0.91 (0.72, 1.15)
CRT+S vs S	**0.77 (0.69, 0.85)**	**0.70 (0.63, 0.78)**	**0.59 (0.53, 0.65)**	0.70 (0.45, 1.08)	0.81 (0.58, 1.12)
RT+S vs S	1.05 (0.95, 1.15)	1.09 (0.99, 1.18)	-	-	-
CRT+S vs CT+S	**0.60 (0.44, 0.75)**	**0.64 (0.49, 0.78)**	**0.74 (0.50, 0.99)**	0.73 (0.44, 1.23)	-

Abbreviation: OS-Overall survival, DFS-Disease-free survival.

Note: The data of 3-year OS, 5-year OS and DFS is HR (hazard ratio) and 95% confidence interval. The results of reccurence and matastasis are OR (odds ratio) and 95% confidence interval.

In conclusion, CT+S and CRT+S had better performance than surgery alone with respect to prognostic indicators, including 3-year OS, 5-year OS and DFS, while CRT+S surpassed the efficacy of CT+S. However, insufficient information provided by a pair-wise meta-analysis and indirect evidence could not be created.

### Network meta-analysis

As for 3-year OS shown in Table 4, CRT+S showed the highest efficacy among the four (CRT+S vs. S: HR=0.81, 95% credential interval (CrI) =0.73-0.90; CRT+S vs. CT+S: HR=0.82, 95% CrI =0.70-0.95; CRT+S vs. RT+S: HR=0.77, 95% CrI =0.62-0.95) and CT+S was inferior to RT+S (HR=0.95, 95% CrI =0.76-1.18) while the two comparisons concerning surgery alone showed no significant statistical difference. Compared to the pair-wise meta-analysis, the network meta-analysis provided us with more comprehensive results such as the comparison between CRT+S and RT+S. Overall, CRT+S acted as the most effective intervention in the treatment of EC with respect to 3-year OS.

**Table 4:** Network meta-analysis results of esophageal cancer treatments

**(a) 3-year Overall Survival**			
**S**	0.94 (0.89, 1.00)	1.08 (0.99, 1.18)	**0.80 (0.74, 0.87)**
1.06 (1.00, 1.13)	**CT+S**	**1.14 (1.02, 1.28)**	**0.85 (0.77, 0.94)**
0.93 (0.84, 1.02)	0.87 (0.78, 0.98)	**RT+S**	**0.74 (0.66, 0.84)**
1.25 (1.15, 1.35)	1.18 (1.07, 1.30)	1.35 (1.19, 1.52)	**CRT+S**
**(b) 5-year Overall Survival**			
**S**	**0.90 (0.85, 0.95)**	**1.10 (1.01, 1.19)**	**0.75 (0.70, 0.81)**
1.11 (1.05, 1.18)	**CT+S**	**1.22 (1.10, 1.35)**	**0.84 (0.77, 0.92)**
0.91 (0.84, 0.99)	0.82 (0.74, 0.91)	**RT+S**	**0.69 (0.62, 0.77)**
1.33 (1.23, 1.43)	1.19 (1.09, 1.30)	1.45 (1.30, 1.62)	**CRT+S**
**(c) Disease-free Survival**			
**S**	**0.81 (0.74, 0.88)**	-	**0.70 (0.65, 0.76)**
1.24 (1.13, 1.36)	**CT+S**	-	**0.87 (0.78, 0.97)**
-	-	**RT+S**	-
1.43 (1.32, 1.54)	1.15 (1.03, 1.29)	-	**CRT+S**
**(d) Recurrence**			
**S**	0.70 (0.29, 1.64)	-	**0.34 (0.11, 0.92)**
1.43 (0.61, 3.46)	**CT+S**	-	0.49 (0.14, 1.58)
-	-	**RT+S**	-
2.95 (1.08, 8.79)	2.05 (0.63, 7.19)	-	**CRT+S**
**(e) Metastasis**			
**S**	0.82 (0.55, 1.15)	-	0.72 (0.44, 1.12)
1.22 (0.87, 1.82)	**CT+S**	-	0.89 (0.48, 1.62)
-	-	**RT+S**	-
1.38 (0.89, 2.25)	1.12 (0.62, 2.08)	-	**CRT+S**

Abbreviation: CRT-chemoradiotherapy, S-surgery, CT-chemotherapy, RT-radiotherapy.

Note: In the overall survival of 3 years and 5 years and the disease-free survival, the data are presented in HR (hazard ratio) and 95% CrI. In the results of reccurence and matastasis, the data are presented in OR (odds ratio) and 95% CrI.

**Figure 3:**
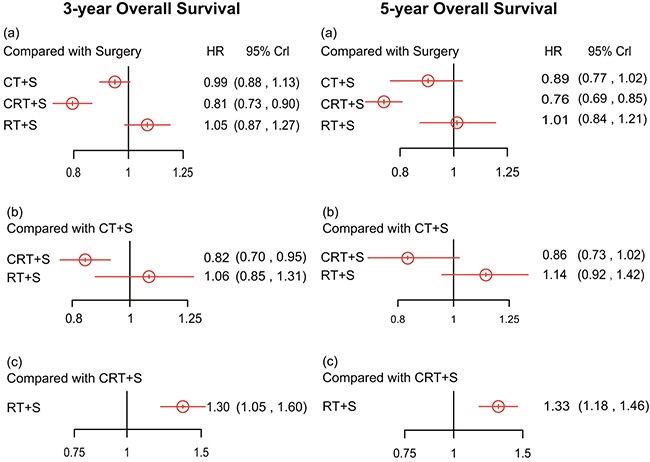
Hazard ratios (95% credential intervals) of overall survival in 3 years and 5 years for network comparison of EC treatments

Likewise, the results from the network meta-analysis with respect to 5-year OS, as displayed in Figure 3, show that all acquired data express a statistical difference. More specifically, CRT+S showed more promising results than all the other three (CRT+S vs. S: HR=0.76, 95% CrI =0.69-0.85; CRT+S vs. CT+S: HR=0.86, 95% CrI =0.73-1.02; CRT+S vs. RT+S: HR=0.75, 95% CrI =0.61-0.93) surgery. The 5-year OS data was almost in accord with 3-year OS, while the comparisons between surgery versus CT+S and surgery versus RT+S showed statistical veracity.

**Figure 4:**
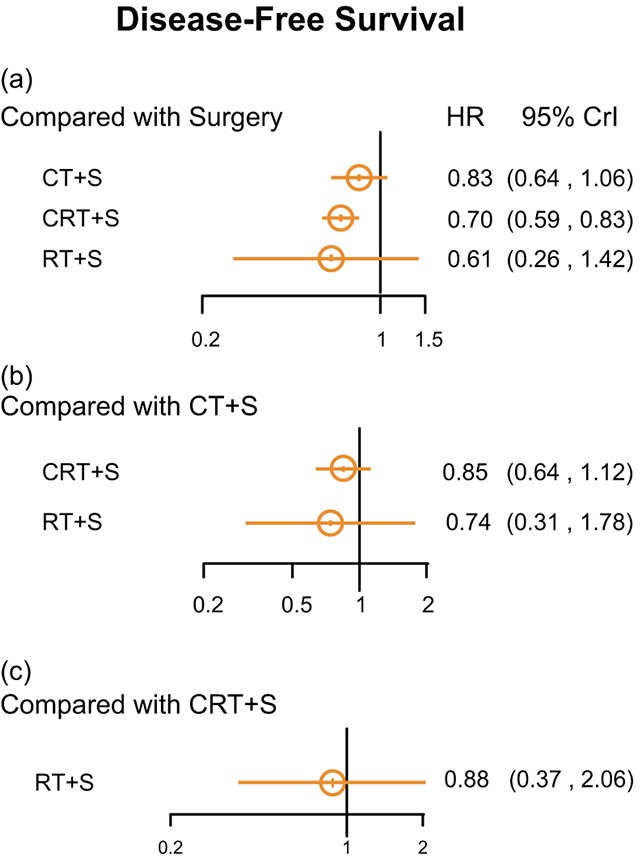
Hazard ratios (95% credential intervals) of disease-free survival for network comparison of EC treatments

Concerning DFS, CRT+S showed an increased efficacy over surgery (HR =0.70, 95% CrI =0. 595-0.83 Figure 4).

**Figure 5:**
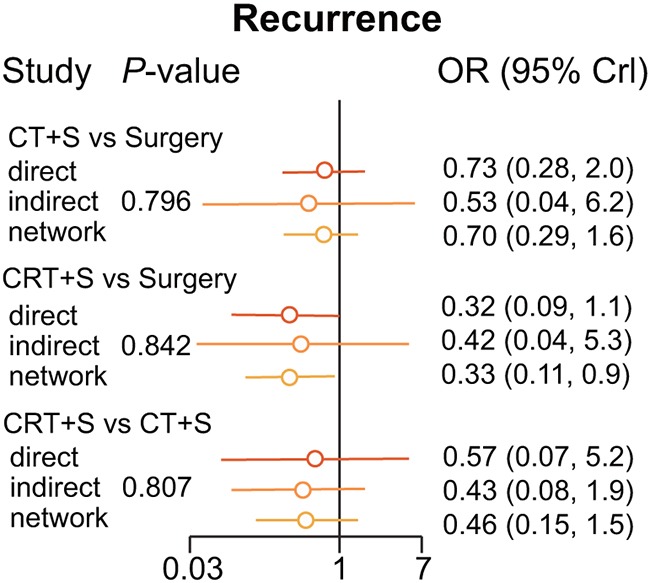
Node Splitting results according to type of treatments for recurrence

**Figure 6:**
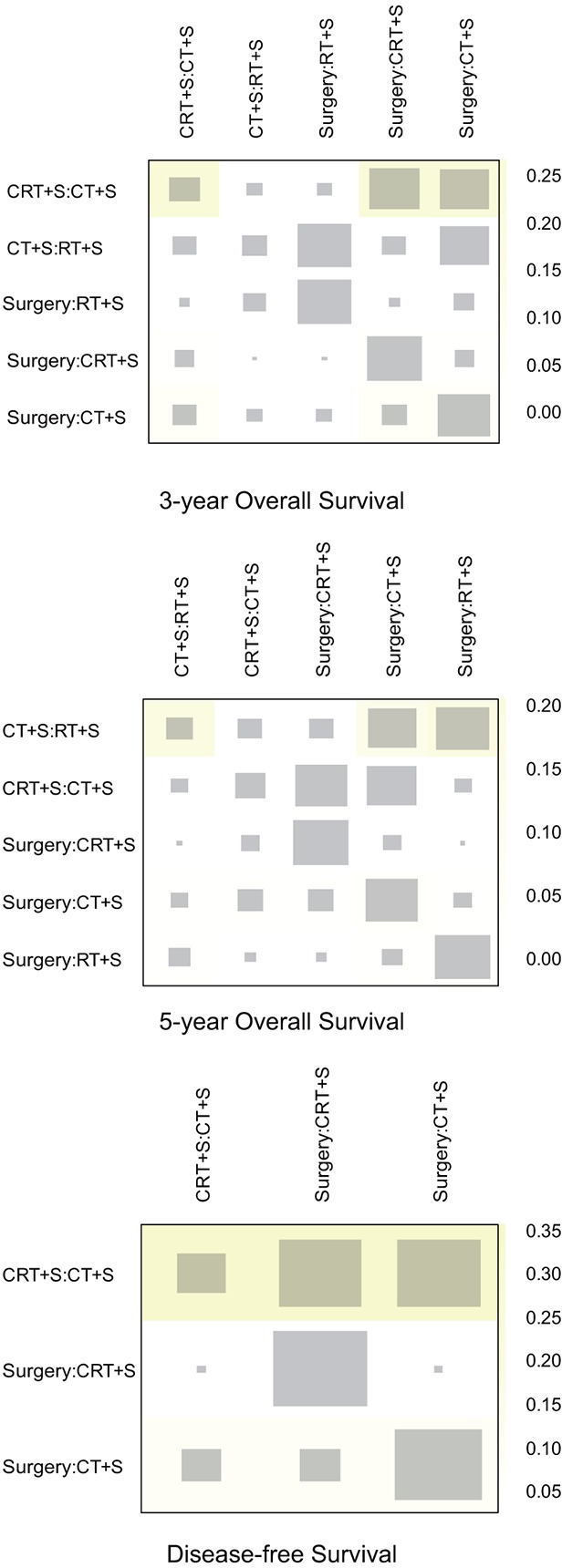
Heat plot for EC treatments The area of the gray squares displays the contribution of the direct estimate in design d (shown in the column) to the network estimate in design d (shown in the row). The colors are associated with the change in inconsistency between direct and indirect evidence (shown in the row) after detaching the effect (shown in the column). Blue colors indicate an increase and warm colors indicate a decrease (the stronger the intensity of the color, the stronger the change).

The only confident result concerning recurrence lay in that CRT+S presented a lower rate of recidivism than common surgery (OR=0.33, 95% CrI=0.11-0.92). Meanwhile, CRT+S demonstrated a superior potential than CT+S (OR=0.46, 95% CrI=0.15-1.50). Such a relation was also seen between CT+S and surgery as shown in the node-splitting results in Figure 5. The heat plot showed the robustness of our results Figure 6.

Finally, Supplementary Table 2 provided the SUCRA values for each strategy and its clinical outcomes. In general, CRT+S ranked best among the tested therapies (surface under the cumulative ranking curve (SUCRA): 3-year OS=0.99; 5-year OS=0.99; DFS=0.76; recurrence=0.93; metastasis=0.80) while surgery alone proved to be the least efficacious treatment concerning recurrence rates (SUCRA=0.11) and metastasis (SUCRA=0.09).

## DISCUSSION

Currently, surgical resection is the preferable treatment for patients without distant metastases (cT_1-3_ N_0-1_ M_0_) [63]. However, the prognoses of patients treated with surgery alone remained poor and could be improved using s adjuvant therapies [21, 63, 64]. Concurrent CRT was found to improve overall survival significantly as well as reduce persistence and recurrence or patients with resectable esophago-gastric adenocarcinoma [64, 65]. Based on previous studies, the advantage of CRT in the treatment of EC has been widely verified, although few studies have been conducted to detect the similarities and differences between other adjuvant therapies and CRT. And according to the current network meta-analysis Pasquali *et al*. [66], CRT+S was the best option, which is consistent to former studies and our results. However, there were some limitations in this study, firstly, no indirect comparison results were provided; besides, the prognosis outcomes were not taken follow-up periods into consideration; additionally, some studies contained duplicate trials such as Natsugoe *et a*l. [60] and Tachibana *et al*. [67].

In our analysis, OS was considered as the primary endpoint. The results showed that CRT+S contributed to the better long-term survival compared with surgery, CT+S and RT+S while RT+S seemed to have no positive effect on survival time, and even resulted in a worsened prognosis. Treatment of CT+S was comparatively superior to surgery and RT+S but inferior to CRT+S with regards to 3-year OS and 5-year OS. The only difference between 3-year OS and 5-year OS was the statistical significance observed between CT+S and RT+S compared to surgery in 5-year OS. The harmful effects of RT+S might be explained by its toxicity to patients. The exposure to RT might lead to acute toxicity which overwhelmed the treatment effects and made the prognosis worse for EC patients [68]. Thus, when it comes to the treatment options, RT alone without any other adjuvant therapies may be considered mainly a palliative tool rather than a curative option for EC patients [69].

DFS was another important endpoint in clinical trials. We detected that CT+S and CRT+S were both beneficial to DFS compared with S, but treatment with CRT+S was better in promoting DFS. Besides survival rate, adverse effects included recurrence and metastasis also compared comprehensively. The results indicated that compared with surgery, CT+S and CRT+S were apt to lower the recurrence and metastasis rates. Furthermore, CRT+S performed better in the prevention of adverse effects ranking first of the treatments analyzed, while CT+S ranked second and surgery third. The results mentioned above are consistent with previous studies [64, 70], which have indicated that CRT+S provides local control of tumors and prevents metastasis. These could be considered reasons for the increased survival rate of patients who have undergone CRT an additional adjuvant treatment.

The results suggest that CRT+S should be the first option taken into consideration, while RT+S should be an option reserved for those who are medically fit for an aggressive modality. Inversely, for patients who are not fit to be exposed to such an aggressive modality, RT may a good choice as a palliative tool. If patients are in the early stages of cancer, therapies with less serious adverse effects are a more pragmatic choice in treatment aimed the eradication of the disease. CRT+S should still be considered a preferred choice in these regards.

However, our study still has several limitations that might affect the interpretation of the results. First of all, the trials on RT are quite rare in our study despite the fact that the study involves a large number of RCTs. And squamous cell cancer is known to be completely different in terms of risk factors, disease biology, tumour location, surgical management from adenocarcinoma of the oesophagus, however we lumped together to provided sufficient data. Additionally, the study includes papers published from 1981 to 2016, spanning 35 years. The time span is long enough that errors are inevitable in our study, considering the development of medical technology. Besides, baselines such as patient clinical stage were not taken into consideration, which may influence the final conclusion. Finally, our study does not take into account the type and dose of chemotherapeutic medications. However, different types and doses of chemotherapeutic drugs do have different effects on patients suffering EC that may result in errors in our analysis.

In summary, we assessed the efficacy and adverse effects of surgery with different adjuvant therapies for EC and drew the conclusion that CTR+S is the most effective option. Patients treated with CRT+S have the best prognosis including long-term survival and low risk of recurrence compared to the other treatments studied here. Furthermore, CT+S is able to reduce adverse effects with an efficacy rate second only to CTR+S. The results of our study may act as guidelines for medical decision and treatment options in the future.

## MATERIALS AND METHODS

### Search strategy

Databases were systematically searched for relevant literature, including PubMed and EMBASE. Key words were used as follows: “esophageal neoplasms”, “surgery”, “chemotherapy”, “radiotherapy”, “chemoradiotherapy”, and “randomized clinical trials”. The results included 3,761 records, 559 were identified as duplicates and hence removed after assessment. 3,153 studies were excluded after identified as irrelevant based on titles and abstracts. Among the 49 studies remaining, full-text articles were reviewed and included if they met the inclusion criteria listed below. This process resulted in 42 studies available and qualified for analysis in this research. They are presented in Table 1.

### Inclusion criteria and data extraction

Articles were included if they: (1) were RCTs with a total of more than 30 samples, had follow-up rates above 90% and follow-up periods of not less than 3 years; (2) contained sufficient information about histology and interventions; (3) provided data on disease-free survival (DFS), 3-year overall survival (OS) and 5-year OS; (4) had at least one pair-wise comparison among surgery alone, or surgery combined with RT, CT and CRT.

The data in Table 1 was extracted from the eligible studies, including the country in which the study was performed, sample size of the intervention and control groups, as well as histology and clinical outcomes. After two investigators reviewed the manuscripts of all the studies independently, the data were extracted into a database. A joint review of the manuscript was performed to solve disagreements until a consensus was reached.

### Statistical analysis

Initially a conventional pair-wise meta-analysis was carried out directly. For each study, HR and then merged the data obtained to discern the overall impact level. The impact level was considered significant if the corresponding 95% CI exceeded 1.

We also performed a network meta-analysis for each endpoint within a Bayesian framework using R 3.2.3 software. The treatment effects were compared through direct and indirect evidence by using HRs or ORs with 95% CrI. Moreover, clinical outcomes such as 3-year OS and 5-year OS were evaluated to estimate the efficacy of the respective treatment. The SUCRA was then used to create a ranking scale of the treatment interventions. For each outcome, the efficacy of a certain intervention was more desirable if a larger SUCRA value was obtained.

## SUPPLEMENTARY MATERIALS FIGURES AND TABLES



## Abbreviations

EC: esophageal cancer
RT: radiotherapy
CT: chemotherapy
CRT: chemoradiotherapy
SUCRA: surface under the cumulative ranking curve
OS: overall survival
