# Supplementary material for: Prognosis of surgery combined with different adjuvant therapies in esophageal cancer treatment: a network meta-analysis
Source: Oncotarget. 2017 Mar 14;8(22):36339–53. doi: 10.18632/oncotarget.16193 (PMC5482659; doi:10.18632/oncotarget.16193)
Supplement: Supplementary file 1 [file oncotarget-08-36339-s001.pdf]

# Prognosis of surgery combined with different adjuvant therapies in esophageal cancer treatment: a network meta-analysis

## SUPPLEMENTARY MATERIALS

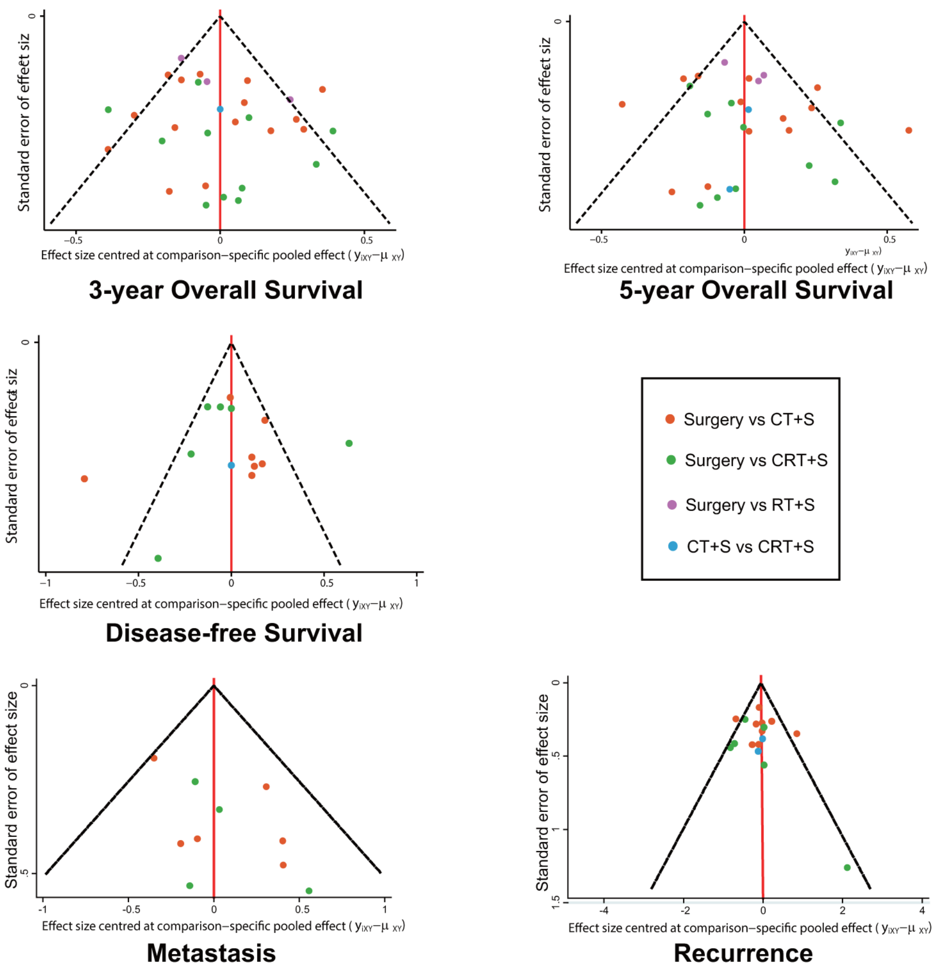


**Supplementary Figure 1: Publication bias of overall survival in 3 years and 5 years, disease-free survival, recurrence and metastasis.**

**Supplementary Table 1: Jadad Scale of 34 included studies**

| Study or Subgroup | Design | Blinding | Withdrawal |
|---|---|---|---|
| Law *et al.,* 1997 | 1 | 0 | 1 |
| Ancona *et al.*, 2001 | 2 | 0 | 1 |
| Kelsen *et al.*, 2007 | 2 | 1 | 1 |
| Allum *et al.*, 2009 | 2 | 1 | 1 |
| Boonstra *et al.*, 2011 | 2 | 2 | 1 |
| Ando *et al.*, 2012 | 1 | 0 | 1 |
| Maipang *et al.*, 1994 | 1 | 0 | 1 |
| Nygaard *et al.*, 1992 | 1 | 0 | 1 |
| Schlag *et al.*, 1992 | 1 | 0 | 1 |
| Ychou *et al.*, 2011 | 2 | 0 | 1 |
| Pouliquen *et al.*, 1996 | 2 | 0 | 1 |
| Ando *et al.*, 1997 | 1 | 0 | 1 |
| Ando *et al.*, 2003 | 1 | 0 | 1 |
| Lee *et al.*, 2005 | 0 | 0 | 1 |
| Heroor *et al.*, 2003 | 0 | 0 | 1 |
| Shiozaki *et al.*, 2004 | 0 | 0 | 1 |
| Zhang *et al.*, 2008 | 0 | 0 | 1 |
| Walsh *et al.*, 1996 | 1 | 0 | 1 |
| Urba *et al.*, 2001 | 1 | 0 | 1 |
| Stahl *et al.*, 2009 | 2 | 0 | 1 |
| Burmeister *et al.*, 2011 | 2 | 0 | 1 |
| Tepper *et al.*, 2008 | 1 | 0 | 1 |
| van Hagen *et al.*, 2012 | 1 | 0 | 1 |
| Burmeister *et al.*, 2005 | 2 | 0 | 1 |
| Lv *et al.*, 2010 | 1 | 0 | 1 |
| Apinop *et al.*, 1994 | 1 | 0 | 0 |
| Le Prise *et al.*, 1994 | 1 | 0 | 1 |
| Walsh *et al.*, 1995 | 1 | 0 | 1 |
| Mariette *et al.*, 2014 | 2 | 0 | 1 |
| Kobayashi *et al.*, 2000 | 2 | 0 | 1 |
| Launois *et al.*, 1981 | 1 | 0 | 1 |
| Gignoux *et al.*, 1987 | 2 | 1 | 1 |
| Arnott *et al.*, 1992 | 2 | 1 | 1 |
| Lee *et al.*, 2004 | 2 | 1 | 1 |

**Supplementary Table 2: SUCRA of different treatments**

| Treatment | 3-year OS | 5-year OS | DFS | Recurrence | Metastasis |
|---|---|---|---|---|---|
| **S** | 0.24 | 0.26 | 0.08 | 0.11 | 0.09 |
| **CT+S** | 0.53 | 0.65 | 0.45 | 0.47 | 0.61 |
| **CRT+S** | 0.99 | 1.00 | 0.97 | 0.93 | 0.80 |
| **RT+S** | 0.24 | 0.09 | - | - | - |

Abbreviation: OS-Overall survival, DFS-Disease-free survival, CRT-chemoradiotherapy, S-surgery, CT-chemotherapy, RT-radiotherapy
